# Development and validation of a pyradiomics signature to predict initial treatment response and prognosis during transarterial chemoembolization in hepatocellular carcinoma

**DOI:** 10.3389/fonc.2022.853254

**Published:** 2022-10-17

**Authors:** Jie Peng, Fangyang Lu, Jinhua Huang, Jing Zhang, Wuxing Gong, Yong Hu, Jun Wang

**Affiliations:** ^1^ Department of Oncology, The Second Affiliated Hospital, GuiZhou Medical University, Kaili, China; ^2^ Department of Minimal Invasive Interventional Therapy, Sun Yat-Sen University Cancer Center, State Key Laboratory of Oncology in South China, Guangzhou, China; ^3^ Department of Medical Imaging Center, Nanfang Hospital, Southern Medical University, Guangzhou, China; ^4^ Department of Oncology, Zhuhai Hospital Affiliated with Jinan University, Jinan University, Zhuhai, China; ^5^ Department of Oncology, Guiyang Public Health Clinical Center, Guiyang, China; ^6^ Department of Oncology, The Third Affiliated Hospital, GuiZhou Medical University, Duyun, China

**Keywords:** epatocellular carcinoma, therapy response, pyradiomics, TACE, progression-free survival

## Abstract

We aimed to develop and validate a pyradiomics model for preoperative prediction of initial treatment response to transarterial chemoembolization (TACE) in patients with hepatocellular carcinoma (HCC). To this end, computed tomography (CT) images were acquired from multi-centers. Numerous pyradiomics features were extracted and machine learning approach was used to build a model for predicting initial response of TACE treatment. The predictive accuracy, overall survival (OS), and progression-free survival (PFS) were analyzed. Gene Set Enrichment Analysis (GSEA) was further used to explore signaling pathways in The Cancer Genome Atlas (TCGA)-HCC cohort. Overall, 24 of the 1,209 pyradiomic features were selected using the least absolute shrinkage and selection operator (LASSO) algorithm. The pyradiomics signature showed high predictive accuracy across the discovery set (AUC: 0.917, 95% confidence interval [CI]: 86.93-96.39), validation set 1 (AUC: 0.902, 95% CI: 84.81-95.59), and validation set 2 (AUC: 0.911; 95% CI: 83.26-98.98). Based on the classification of pyradiomics model, we found that a group with high values base on pyramidomics score showed good PFS and OS (both *P*<0.001) and was negatively correlated with glycolysis pathway. The proposed pyradiomics signature could accurately predict initial treatment response and prognosis, which may be helpful for clinicians to better screen patients who are likely to benefit from TACE.

## Introduction

Hepatocellular carcinoma (HCC) is a major cause of cancer-related death worldwide (1). Some patients with HCC are ineligible for liver transplantation and surgical resection because the curative surgery cannot be performed (2–4). For these patients with intermediate and advanced-stage HCC, transarterial chemoembolization (TACE) therapy is a promising treatment method following the National Comprehensive Cancer Network (NCCN) clinical practice guideline (5–7).

Initial treatment response has recently been reported to be a powerful indicator of favorable outcomes, such as longer progression-free survival (PFS) and overall survival (OS) (8–10). Studies on tumor burden were evaluated using magnetic resonance imaging (MRI) or computed tomography (CT) and found associations between imaging features (e.g., tumor size, tumor number) and treatment response to TACE in patients with HCC (11–14). However, imaging features have a limited accuracy of subjective judgment, and they do not reflect intra-tumor heterogeneity. Developing a robust and accurate algorithm to select patients who will show initial response to treatment remains challenging and important. Thus, an accurate model to identify patients with an initial response to TACE could be useful to optimize individualized treatment strategy.

Nowadays, radiomics have been a new and promising field that involves the extraction of large quantitative features from radiographic images (15, 16). The radiomics algorithm offers an unprecedented opportunity to improve cancer decision-making in a low-cost and non-invasive manner. Previous studies have shown that radiomics models of radiology images are significantly associated with clinical outcomes in cancer patients (17–21). We previously found that a radiomics model based on CT images could precisely predict microvascular invasion in HCC patients and the machine learning algorithm could be used to predict clinic outcome in cancer (22, 23). However, the standard method in this field was lacked and the potential mechanism of radiomics model was unclear in the HCC. Radiomics extracting from python package was named pyradiomics and provide the chance. The role of pyradiomics predictive models for initial response or prognosis in TACE treatment, and the association with signal transduction pathway remain unexplored.

In this study, based on the preoperatively CT images and machine learning algorithm, we aimed to develop and validate a robust and accurate pyradiomics signature for a noninvasive pretreatment prediction of initial response to TACE in HCC patients. The mutual relationships between pyradiomics score and clinical factors were further analyzed and validated in other patient cohorts. The subgroup analyses, clinical utility and prognosis of pyradiomics model were estimated. Gene Set Enrichment Analysis (GSEA) tool was used to reveal the association between pyradiomics model and Kyoto Encyclopedia of Genes and Genomes (KEGG), which contributes to interpretation of the potential mechanism but not “black box” of these machine learning models.

## Materials and methods

### Study design and patients

**Figure 1:**
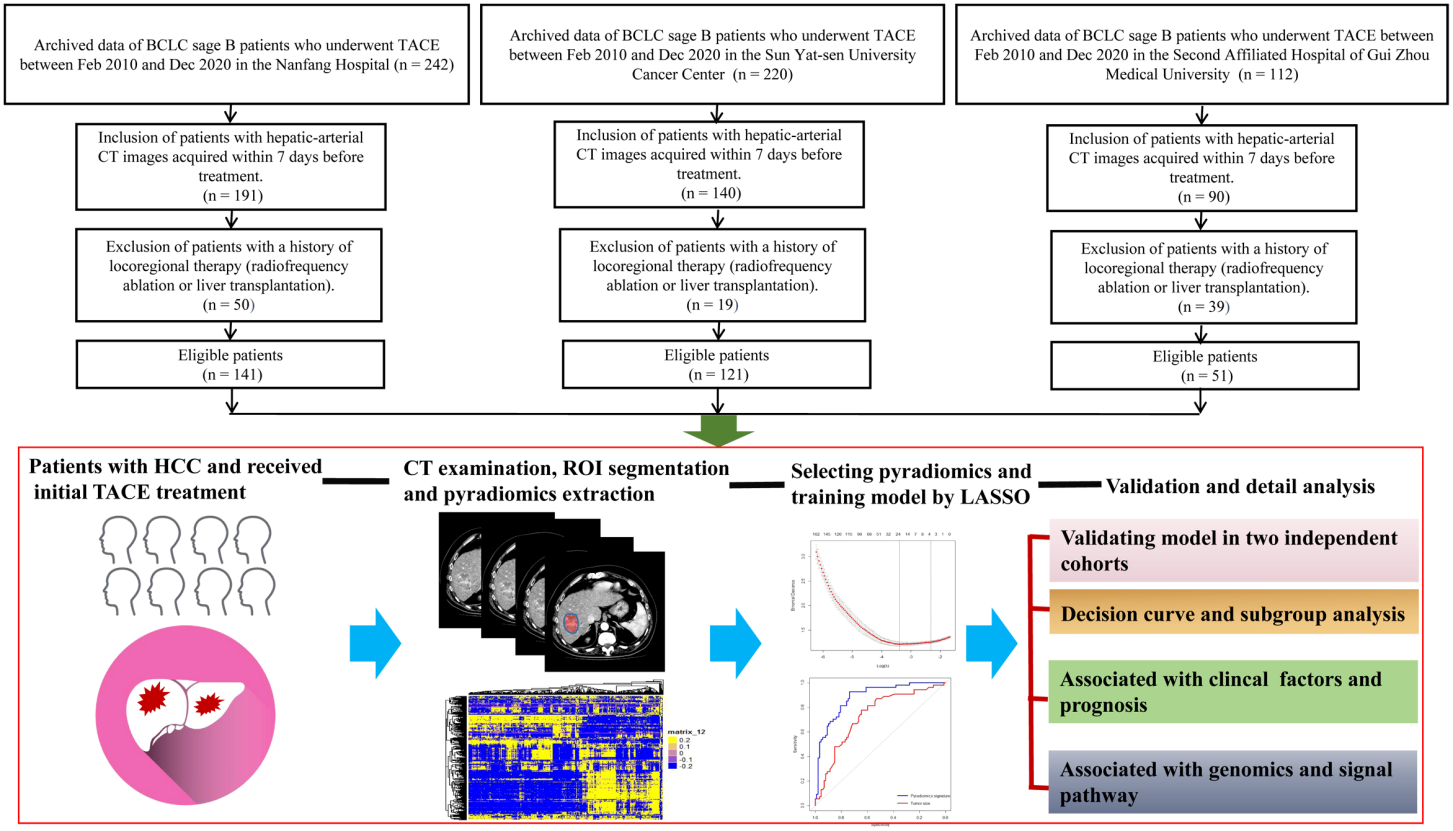
Flowchart for the development of the machine learning model. All patients have radiologically or pathologically proven HCC and undergo CT before TACE therapy. The radiologists manually segment the 3D ROIs. Thereafter, 1,167 features are extracted from the hepatic arterial CT images based on the “pyradiomics” package of python. Using LASSO method, 24 features are selected, and the pyradiomics model is built. Model validity is evaluated in two cohorts. The predictive performance of the pyradiomics model is compared with that of clinical factors. The association between the pyradiomics model and cancer signaling pathways is analyzed using the TCGA-HCC cohort. 3D, three-dimensional; HCC, hepatocellular carcinoma; CT, computed tomography; ROI, region of interest; LASSO, least absolute shrinkage and selection operator algorithm; TCGA, The Cancer Genome Atlas.

The flowchart of the machine learning model is presented in detail in **Figure 1**. This was a retrospective study of 313 patients with Barcelona Clinic Liver Cancer (BCLC) stage B HCC who underwent conventional TACE between February 2010 and December 2020. Patients were recruited from the Nanfang Hospital (n=141 patients, discovery set), Sun Yat-sen University Cancer Center (n=121, validation set 1), and the Second Affiliated Hospital of Gui Zhou Medical University (n=51) (validation set 2). The inclusion criteria were radiologically or pathologically confirmed HCC, initial TACE treatment, BCLC sage B, and arterial-phase CT images availability within 7 days before and 30 days after treatment. Patients who underwent loco-regional or whole-body therapies were excluded. According to the modified Response Evaluation Criteria in Solid Tumors (mRECIST), the initial response to TACE was classified as complete response (CR), partial response (PR), stable disease (SD), and progressive disease (PD) by an experienced radiologist as in the previous study (24). Initial treatment response and non-response were strictly defined as CR+PR and SD+PD, respectively. The OS was defined as the time from the start of TACE or hepatectomy treatment until death or the last contact; PFS was defined as the time between the beginning of TACE treatment and the progression or death of the tumor; disease-free survival (DFS) was defined as the time between the beginning of hepatectomy treatment and disease recurrence or death because of the tumor. This study was approved by the three institutional review boards of Nanfang Hospital, the Second Affiliated Hospital of Gui Zhou Medical University, and Sun Yat-sen University Cancer Center.

### TACE procedure, CT acquisition and manual segmentation of the region of interest

TACE was performed under local anesthesia using the traditional femoral approach. TACE was performed under the guidance of digital subtraction angiography (Allura Xper FD 20, Philips) through the left and right hepatic arteries directly through the arteries supplying blood to the tumor when technically feasible. Hepatic arteriography, performed using a 5 Fr (RH or Yashiro) catheter, was first used to assess the location, number, size, and blood supply of the target tumor. The embolic emulsion agent, including epirubicin (30–60 mg), lobaplatin (30–50 mg), and lipiodol (10–30 mL), was injected into the artery supplying the tumor through a 2.7/2.8 Fr microcatheter. Thirty days after treatment, according to the modified Response Evaluation Criteria in Solid Tumors (ver. 1.1).

Contrast-enhanced computed tomography scans were performed as previously described (22). Contrast-enhanced computed tomography (CECT) was performed at hospital using an MDCT scanner and the detail information of CT image acquisition was described in the **Supplementary Material**. After the routine CT scanning, a contrast agent (Ultravist 370, Bayer SchL/s) was delivered *via* an injector (Ulrich CT Plus 150, Ulrich Medical, Ulm, Germany); and CECT was performed immediately after injection. Preoperative CT images were collected on the Picture Archiving and Communication System (PACS; Nanfang Hospital Network Center, China), with an optimal window setting adjustment (window width: 300, window level: 50). The CT images were downloaded through the Picture Archiving and Communication System. Two senior radiologists blinded to the treatment results manually segmented the three-dimensional (3D) regions of interest (ROI) in HCC using the ITK-SNAP (version 3.6, https://sourceforge.net/projects/itksnapx64/). Then, they saved and stored the main images and 3D segmented images for extraction of pyradiomics.

### Extraction and reproducibility examination of pyradiomics features

MATLAB 2014b (https://ww2.mathworks.cn/) was used to standardize and reconstruct the segmented 3D ROI image. The thickness of the layer was 1 mm. Python 3.6 (https://www.python.org/downloads/release/python-360/) was used to install the package (https://github.com/Radiomics/pyradiomics) and extract the pyradiomics features from 3D images. These values included the texture, shape, size, and wavelet transform of the CT images. The intra- and inter-correlation coefficients (ICCs) of 50 hepatic artery CT images between two observers were used to evaluate the repeatability of pyradiomics feature extraction. To evaluate intra-observer reproducibility, two radiologists independently and manually segmented the ROIs of 50 patients. Meanwhile, to assess repeatability between observers, two readers extracted the high-dimensional pyradiomics features twice with at least 1-week interval. ICCs greater than 0.75 were set to indicate favorable consistency in pyradiomics extraction. These values, which described the texture, shape, size, and wavelet transform of the CT images, could be used to analyze the overall consistency and select the robust pyradiomics features with good reproducibility.

### Development and validation of the pyradiomics signature for predicting therapy response

The least absolute shrinkage and selection operator (LASSO) is a powerful algorithm to choose the most important variables from high-dimensional features (22, 23). LASSO is a reduction method that shrinks the regression coefficient to a certain area. The main idea in using LASSO is constructing a first-order penalty function to obtain a refined model through the final determination of some variables coefficient 0 for feature screening. The penalty term of LASSO is:


$$\sum_{i=1}^{n}|wi|\leq t$$

This constraint uses the first-order penalty function of absolute value instead of the second-order function of square sum. Although the form is only slightly different, the results are very different. Some of the coefficients would generate to zero. In this study, LASSO based on 5-fold cross-validation was used to select 24 non-zero coefficients. Then, a pyradiomics score (PRS) was calculated based on a logistic method. A pyradiomics signature was consequently developed to predict TACE treatment response. The pyradiomics model’s performance was then evaluated in the discovery and two validation sets using receiver operating characteristic (ROC) analysis. The optimal cut-off value for predicting treatment response was calculated using the Youden’s index. According to the optimal cut-off PRS value, we divided the patients into two groups. The patients with high values (>-0.14) were defined as RS1, and those with low values (≤-0.14) as RS2. In our study, 112 patients from Nanfang Hospital and 29 from the Second Affiliated Hospital of Gui Zhou Medical University had the information of prognosis.

### Gene Set Enrichment Analysis in the TCGA-HCC cohort

The purpose of Gene Set Enrichment Analysis (GSEA) is to explore the relationship between the level of imaging score and tumor-related signaling pathways and gene expression. The GSEA analysis data included four files: 1. The Gene Expression profiling data (Expression dataset) was derived from the mRNA Expression profiles of HCC in The Cancer Genome Atlas (TCGA), and contained 20,533 Gene Expression information; 2. The Gene sets contained 2,074 known Gene sets; 3. Chip annotations listed each probe on the DNA Chip and its matching Hugo gene symbols; 4. Phenotype labels were used to categorize samples into two classes for research purposes and to ensure that the order of the samples was consistent with that of the expression spectrum files. TCGA expression spectrum data were downloaded at: https://www.cancer.gov/about-nci/GDC. The data for a total of 46 HCC patients with preoperative CT images in TCGA database were downloaded from The Cancer Imaging Archive (TCIA) (http://www.Cancer.imaging.archive.net/). In this study, according to the value of PRS, 46 patients with HCC were divided into two groups: RS1 and RS2. The two groups were clustered to identify the distinct genes (fold change ≥2.0, *P*<0.05). Genes were identified using the “edgR” package. Based on the specific genes, pathway analysis was conducted to determine the potential mechanisms for the machine learning model (DAVID, https://david.ncifcrf.gov/).

### Statistical analysis

The “pROC” package was used to plot the receiver operating characteristic (ROC) curves. A confidence interval (CI) of 95% for the area under the curve (AUC) was calculated in all cohorts. PRS was evaluated using the Mann-Whitney U test. The Akaike information criterion (AIC) was used to select the optimal model. The AIC is based on entropy and a measure of the goodness of a statistical model. The smaller the AIC, the better the model. The AIC can be expressed as: AIC = (2k-2L)/n. The Kaplan-Meier curves of DFS, PFS, and OS were analyzed using the “survminer” package. Decision curve analysis (DCA) was used to quantify the probabilities of net benefits at different threshold in patients with HCC, plotted by the “dca.R” package. All statistical analyses were performed using the R statistical software version 3.5.0 (R Core Team, 2018) and GraphPad prism 7.0. Two-sided *P* values<0.05 were considered significant.

## Results

### Patient characteristics

In total, 18 (12.76%), 13 (10.75%), and 10 (19.61%) patients in the training set, validation set 1, and validation set 2 were females, respectively. Furthermore, 93 (65.96%), 85 (70.25%), and 33 (64.70%) patients were aged less than 60 years, respectively. The baseline patient characteristics are shown in **Table 1**. Most patients (82.27, 86.78, and 82.35%) had Child-Pugh A disease and a low number of tumors (≤3) (85.82, 86.64, and 76.47%) in the training set, validation set 1, and validation set 2, respectively. Overall, 51.06, 52.06, and 49.01% of the patients in the training set, validation set 1, and validation set 2, respectively, had high alpha-fetoprotein (AFP) levels. Patients with small tumor size (≤5 cm) accounted for 13.47, 12.39, and 15.69% of the population in the discovery set, validation set 1, and validation set 2, respectively. There were 56 (39.72%), 51 (42.15%), and 19 (37.25%) patients in these three cohorts, respectively, who achieved CR/PR. There was no significant difference in the treatment response rate between the three sets.

**Table 1:** Patient characteristics by study set.

Variable	Discovery set (n = 141)	Validation set 1 (n = 121)	Validation set 2 (n = 51)	*P* value
Sex				0.286
Female	18 (12.7%)	13 (10.7%)	10 (19.6%)	
Male	123 (87.3%)	108 (89.3%)	41 (80.4%)	
Age (years)				0.687
≤60	93 (65.9%)	85 (70.3%)	33 (64.7%)	
>60	48 (34.1%)	36 (29.7%)	18 (35.3%)	
Child–Pugh classification				0.573
A	116 (82.3%)	105 (86.7%)	42 (82.3%)	
B	25 (17.7%)	16 (13.3%)	9 (17.7%)	
AFP (ng/mL)				0.935
≤20	72 (51.1%)	63 (52.1%)	25 (49.1%)	
>20	69 (48.9%)	58 (47.9%)	26 (50.9%)	
Tumor size (cm)				0.967
≤5	19 (13.5%)	15 (12.4%)	8 (15.7%)	
>5, ≤10	62 (44.0%)	57 (47.1%)	23 (45.1%)	
>10	60 (42.5%)	49 (40.5%)	20 (39.2%)	
Tumor number				0.915
≤3	121 (85.8%)	100 (86.6%)	39 (76.5%)	
>3	20 (14.2%)	21 (13.4%)	12 (23.5%)	
Treatment response				0.823
CR/PR	56 (39.7%)	51 (42.2%)	19 (37.3%)	
SD/PD	85 (60.3%)	70 (57.8%)	32 (62.7%)	

P value is derived from the difference between the discovery data set and the two validation data sets. AFP, alpha-fetoprotein; CR, complete response; PR, partial response; SD, stable disease; PD, progressive disease.

### Pyradiomics signature development and associated analysis of clinical factors

Pyradiomics features were extracted as described in previous studies (25–27). A total of 1,167 features were extracted from the hepatic arterial 3D-CT images. A total of 457 pyradiomics features were eliminated in the ICC analysis. The remaining 710 features were then subjected to feature selection and LASSO coefficient analysis. Based on 5-fold cross-validation *via* the maximum criteria, 24 coefficients were selected (**Supplemental Figure S1**). Twenty-four pyradiomics features were analyzed *via* multi-variable logistic regression and included to develop the pyradiomics signature (PRS) (**Table 2**).

**Table 2:** Formula for calculating pyradiomics signature.

Pyradiomic features	Coefficients	*P* value
Intercept	1.479e+02	0.243
exponential_glrlm_Long Run Emphasis	2.502e+04	0.989
exponential_glrlm_Long Run Low Gray Level Emphasis	-2.502e+04	0.989
exponential_glszm_Small Area High Gray Level Emphasis	1.976e+01	0.624
logarithm_first order_Skewness	-9.396e-02	0.046*
logarithm_glcm_Idmn	1.004e+02	0.192
original_gldm_Dependence Variance	-5.060e-02	0.145
original_gldm_Small Dependence High Gray Level Emphasis	-3.662e-02	0.818
original_glszm_Gray Level Non Uniformity	-1.305e-04	0.851
original_shape_Maximum 2D Diameter Slice	4.262e-03	0.732
original_shape_Maximum 3D Diameter	6.609e-03	0.559
original_shape_Sphericity	9.891e+00	0.030*
square_glszm_Small Area Emphasis	-1.362e+00	0.115
wavelet.HHL_firstorder_Skewness	6.523e-01	0.159
wavelet.HHL_glcm_Cluster Prominence	3.869e-05	0.660
wavelet.HHL_glszm_Gray Level Non Uniformity	-2.459e-04	0.308
wavelet.HHL_glszm_Large Area High Gray Level Emphasis	-8.487e-11	0.059
wavelet.HHL_glszm_Low Gray Level Zone Emphasis	2.924e+00	0.001*
wavelet.HLH_gldm_Dependence Non Uniformity Normalized	-2.301e+01	0.030*
wavelet.LHH_first order_Skewness	-4.023e-01	0.072
wavelet.LHL_glszm_Large Area Low Gray Level Emphasis	-4.079e-08	0.252
wavelet.LLH_first order_Median	1.630e+00	0.251
wavelet.LLH_glcm_Cluster Shade	-9.764e-01	0.082
wavelet.LLL_first order_90 Percentile	2.615e-03	0.083
wavelet.LLL_glcm_Idmn	-2.532e+02	0.068

*P<0.05 indicates statistical significance.

**Figure 2:**
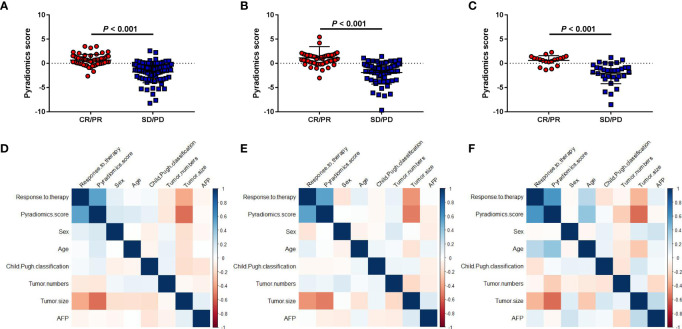
Association between the pyradiomics score and clinical factors. **(A-C)** Correlations between the pyradiomics score and object response (CR+PR) in the discovery and two validation sets. **(D-F)** Correlation heatmaps of the pyradiomics score and clinical factors in the three cohorts. SD, stable disease; CR, complete response; PD, progressive disease; PR, partial response.

Treatment responders showed significantly higher PRS than non-responders in the three cohorts (all *P*<0.001) (**Figures 2A-C**). Tumor size was also associated with treatment response in the three cohorts (*P*<0.001, *P*<0.001, and *P=*0.017, respectively) (**Supplemental Figure S2**). However, we found there was no association between treatment response and other clinical factors, such as tumor number, AFP level. Meanwhile, PRS was also not correlated with age, sex, Child-Pugh classification, AFP level, and tumor number across all three cohorts (**Figures 2D-F**). In contrast, PRS was significantly associated with tumor size (r=-0.416, *P*<0.001; r=-0.514, *P*<0.001; r=-0.568, *P*<0.001, respectively) and treatment response (r=0.605, *P*<0.001; r=0.539, *P*<0.001; r=0.588, *P*<0.001, respectively).

### Evaluating classifiable accuracy of machine learning model by PRS

**Figure 3:**
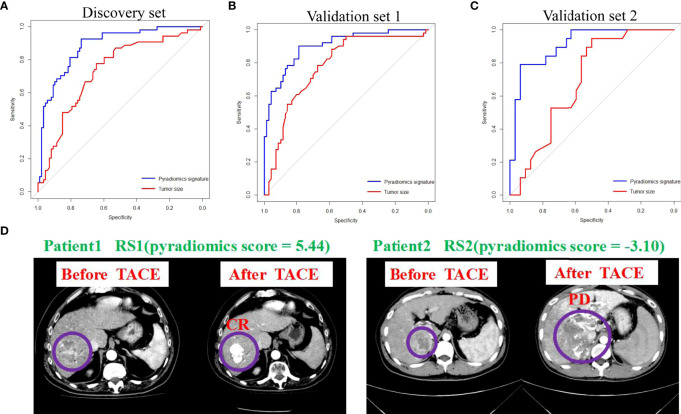
Pyradiomics signature and tumor size predict treatment response to TACE. **(A-C)** ROC curves show the predictive performance of pyradiomics signature and tumor size for estimating CR and PR. The bootstrap (n=2000) test results of the two ROC curves indicate that the AUC of the sum of pyradiomics signature is significantly higher than those of the tumor size in the discovery and in validation 1 and 2 sets. **(D)** Examples of two patients with response (RS1) or no response (RS2). ROC, receiver operating characteristic; AUC, area under curve; CR, complete response; PR, partial response.

The area under the ROC curves of tumor size and the pyradiomics signature were analyzed. We used Youden’s index (defining as sum of sensitivity and specificity minus 1) to calculate the optimal cut-off value (-0.14) in the ROC analysis. The AUCs showed that the tumor size (AUC=0.752, 95% CI: 66.89-83.60, *P*<0.001) and pyradiomics signature (AUC=0.916, 95% CI: 86.93-96.39, *P*<0.001) could be the predictors of treatment response to TACE in patients with HCC (**Figure 3A**) (**Supplemental Table S1**). Furthermore, the model of the pyradiomics signature in the validation sets 1 and 2 also demonstrated a high AUC for predicting treatment response, with the AUC consistent with that in the discovery set (AUC=0.902, 95% CI: 84.81-95.59, *P*<0.001; AUC=0.911, 95% CI: 83.26-98.98, *P*<0.001, respectively). The AUCs of the tumor size were 0.778 (95% CI: 52.00-82.60, *P*<0.001) and 0.690 (95% CI: 54.66-83.33, *P*=0.024, **Figures 3B, C**). Based on boostrap (n=2000) analysis, the efficiency of the pyradiomics signature was significantly higher than that of the tumor size in the discovery (*P*=0.016) and the two validation sets (*P*=0.016 and *P*=0.008). The AIC of the model comprising a combination of the pyradiomics signature and tumor size was not superior to that of the model comprising only the pyradiomics signature in the discovery set (240.53 vs. 238.94). The examples of two patients with response (RS1) or no response (RS2) are shown in our study (**Figure 3D**). The patient 1 had higher pyradiomics score than patient 2 did (5.44 vs.-3.10).

### Clinical utility and subgroup analysis of PRS predictive accuracy

**Figure 4:**
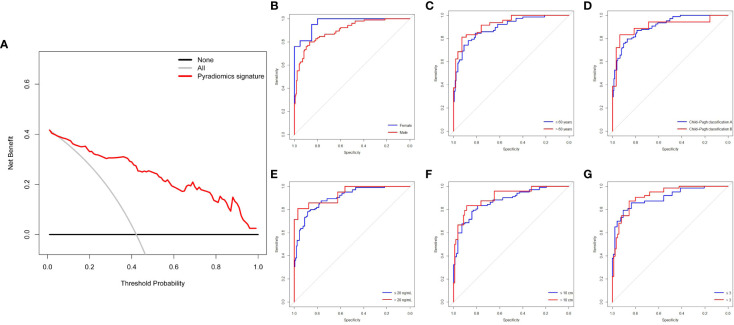
DCA and subgroup analysis of the pyradiomics signature for predictive treatment response to TACE. **(A)** DCA of the pyradiomics signature. **(B-G)** Pyradiomics signature of clinical subgroup predicts therapy response in all patients (n=313) undergoing TACE treatment. The two ROC curves are compared using the bootstrap (n=2000) test. DCA, decision curve analysis; ROC, receiver operating characteristic; TACE, transarterial chemoembolization.

The DCA of the pyradiomics signature showed relatively good performance of the model regarding clinical application (**Figure 4A**). It was suggested from the DCA curve that when the threshold probability in a patient was 42%, more initial response could be achieved through a pyradiomics signature than either treat-all or treat-none strategies. The probability of acquiring treatment response ranged from 8 to 100%. Thus, a pyradiomics signature accurately identifies the patients who have the initial response and may benefit from TACE therapy. The patients were divided into subgroups based on six clinical variables to estimate the classification performance of the pyradiomics model further (**Supplemental Table S2**). The AUC was higher in the female patients than that in the male patients (0.966, 95% CI: 92.18-100.00 vs. 0.8985, 95% CI: 86.12-93.59, *P*=0.021) (**Figure 4B**). Meanwhile, the predictive accuracy was not affected by age, Child–Pugh classification, and AFP levels, compared with bootstrap=2000 (*P*=0.354, *P*=0.998, and *P*=0.424, respectively) (**Figures 4C-E**). Subgroup analysis by tumor number and tumor size also showed no significant difference in AUCs (*P*=0.443 and *P*=0.597, respectively) (**Figures 4F-G**).

### The prognostic value of PRS in patients undergoing TACE treatment

**Figure 5:**
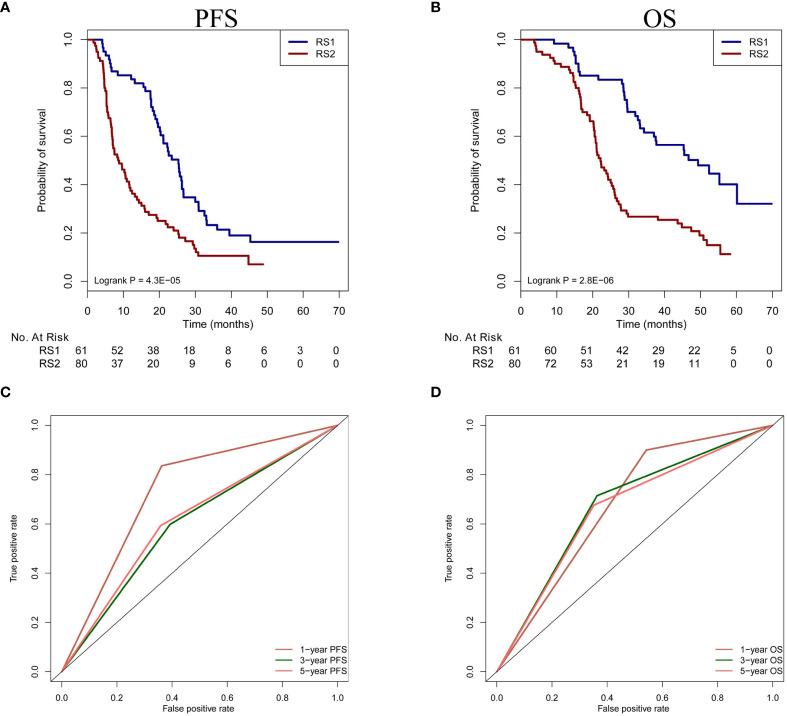
Prognosis prediction of PRS in patients undergoing TACE treatment. **(A, B)** The OS and PFS of two classification (RS1 vs. RS2) are compared in the patients. **(C, D)** Time-dependent ROC curve analysis is performed in 1-year-OS/PFS, 3-year-OS/PFS and 5-year-OS/PFS predictions. PFS, progression-free survival; OS, overall survival; PRS, pyradiomics score; TACE, transarterial chemoembolization; ROC, receiver operating characteristic.

According to the optimal cut-off value of PRS, we divided the patients into two groups. The patients with high values (>-0.14) defined as RS1, and the patients with low values (≤-0.14) defined as RS2. In our study, 112 patients from Nanfang Hospital and 29 patients from the Second Affiliated Hospital of Gui Zhou Medical University had the information of prognosis. Therefore, based on stratification of RS1and RS2, PFS and OS were analyzed in a total of 141 patients. We found the patients with RS1 had a longer PFS and OS than those with RS2 (Median PFS: 25 vs. 9 months, hazard ratio [HR]=2.78, 95% CI: 1.93-4.00, *P*<0.001; Median OS: 49 vs. 22 months, HR=2.23, 95% CI: 1.47-3.38, *P*<0.001, respectively) (**Figures 5A, B**). Moreover, we used the biomarkers of RS1 and RS2 to predict the 1-, 3-, and 5-year-OS and PFS rates. In the time-dependent ROC curve estimation from censored survival, the higher accuracies were 1-year-OS/PFS predictions than 3- and 5-year-OS/PFS predictions (0.679 vs.0.676 vs.0.662; 0.737 vs. 0.602 vs.0.617, respectively) (**Figures 5C, D**).

### Association between PRS and glycolysis pathway genes stratify prognosis in TCGA-HCC patients (No-TACE treatment)

**Figure 6:**
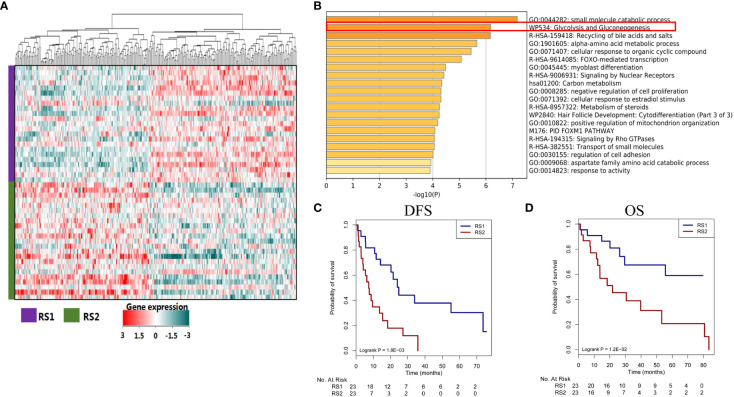
Different genes, signaling pathways, and prognosis associated with predicted treatment response. **(A)** The heat map plot of mRNA expression from different genes. Patients with HCC are shown on the *y*-axis, and gene expression is shown on the *x*-axis. **(B)** Some genes are enriched in certain signaling pathways, such as in the glycolysis and gluconeogenesis pathways. **(C, D)** The responder group shows better DFS and OS than the non-responder group does. HCC, hepatocellular carcinoma; DFS, disease-free survival; OS, overall survival.

The same algorithm of machine learning in the training cohort was used in the CT images of 46 patients with HCC from the TCGA database (TCGA-HCC cohort) and the PRS was calculated by the pyradiomics formulation. According to the above cut-off value of pyradiomics score, all patients were then divided into two groups, which were defined as RS1 group (n=23) and RS2 group (n=23). In this experiment, high pyradiomics scores were mostly observed in the RS2 group and low pyradiomics scores were contrary. Through differential gene expression analysis (RS1 vs. RS2), between-group comparisons showed that 151 genes were significantly downregulated, while 167 genes were upregulated (FDR adjust *P*<0.05) (**Figure 6A**) (**Supplemental Figure S3**). The GSEA found several KEGG pathways were significantly associated with PRS, such as small molecule catabolic process, glycolysis, and recycling of bile acids and salts. In many pathways, we speculated that glycolysis resulting from tumor hypoxia status were mostly associated with TACE treatment. Therefore, we mainly focused on the glycolysis pathway genes and found that RS1 group was negatively associated with *HK2* and *PFKP* presence (**Figure 6B**) (**Supplemental Figure S4**). We cound not determine the ability of the PRS in this study to evaluate clinical prognosis in patients with HCC who underwent hepatectomy but did not receive TACE therapy. We subsequently found that RS1 group had significantly longer DFS and OS time than the RS2 group did (*P*<0.001 and *P*=0.008, respectively) (**Figures 6C, D**).

## Discussion

Based on preoperatively CT images, this study developed a pyradiomics signature to accurately predict the initial treatment response and prognosis in HCC patients who underwent TACE therapy. The DCA and subgroup prediction analysis showed the good clinical performance of the model. We further analyzed the association between the pyradiomics model and KEGG pathway genes in TCGA-HCC database. Our findings provided a novel insight into the interpretability of the machine learning model for predicting therapy response or prognosis in different types of tumors.

TACE is a standard treatment modality for HCC patients with BCLC stage B disease (7). Although recent studies reported that there was a non-superiority of TACE with respect to bland embolization, and direct incremental costs of drug-eluting beads TACE (DEB-TACE) can be acceptable in hepatocarcinoma patients (28, 29), considering the extensiveness of method and health policy in our country, we chose the conventional TACE treatment in this study. Treatment response to the first TACE is a well-known predictor of clinical outcomes in patients with middle-stage HCC. This study used pyradiomics algorithms from the python package to extract features of tumor shape, texture, intensity, and wavelet transform characteristics from three-dimensional CT images in HCC patients who underwent TACE as reported by previous studies (30, 31). In our study, we used LASSO of 5-fold cross-validation to chose pyradiomics features for CT images, and 24 features were finally selected. We then used logistic regression analysis to calculate the pyradiomics score among HCC patients who underwent TACE. The results showed significantly higher pyradiomics scores in the response group. The response group also showed smaller tumor size, which was consistent with previous reports (32, 33). There was a significantly negative correlation between the pyradiomics score and tumor size. Compared with tumor size, the pyradiomics model showed higher accuracies for predicting treatment response. This could be because the pyradiomics model included larger amount of radiology information than the model based on tumor size did (34), and a machine learning method, such as LASSO, made a great contribution to enhance the predictive ability of diagnosis, therapy response or prognosis in solid tumors. However, the model combining the pyradiomics signature and tumor size did not show superior AIC to that using the pyradiomics signature alone. The machine learning model had a robust accuracy for predicting initial treatment response to TACE and the method could be used as an invasive stool in the other cancers.

The DCA revealed that the pyradiomics model could predict the treatment response when the probability of acquiring treatment response ranged from 8 to 100%. This result indicated that the pyradiomics signature could help determine clinical strategy and identify the patients who had initial treatment response according to the above the optimal cut-off value (>-0.14). Additionally, some clinical variables may affect the predictive accuracy of the pyradiomics model (6, 35), and the model’s predictive accuracy in patient subgroups was rarely reported and was still unclear. Interestingly, in this study, our subgroup analysis (**Supplemental Table S2**) showed that our machine learning model had a better predictive accuracy in females than in male patients. Of note, the sample size of female patients was significantly smaller than that of male patients (41 vs. 272). The different result of TACE treatment in male or female could be further confirmed by large patients. Meanwhile, subgroup analysis by age showed no significant difference in accuracies between patients aged ≤60 years and >60 years. There were also no significant differences in accuracy according to the Child–Pugh classification, AFP, tumor size, and number of tumors. Collectively, these results support that our pyradiomics model can accurately predict individual treatment response to TACE and may help identify patients who will benefit from the treatment. In addition, the correlation between predictive classification of pyradiomics model and prognosis was investigated. We found the RS1 group indicating well response of initial TACE treatment had better OS and PFS than RS2 group did. This result further demonstrated the initial response could improve the OS and PFS in the patients receiving TACE therapy. Moreover, our model based on the series CT images and analysis of time-dependent ROC curve could preoperatively predict the prognosis and to screening of patients who could benefit from TACE treatment among the BCLC stage B patients with HCC.

To explore the potential mechanism of the pyradiomics model, we used CT images from the TCGA-HCC cohort to screen the different genes and related cancer signaling pathways (**Supplemental Figure S3**). RS1 group was significantly associated with hypoxia. Previous studies have reported that the tumor hypoxia status is associated with resistance to chemotherapy, targeted therapy, and radiation therapy (36–38). This indicates that our machine learning model predicts treatment response to TACE by characterizing the hypoxia change in tumors from CT images. In the TCGA-HCC patients (No-TACE treatment), RS2 group with severe hypoxia also showed a significantly poorer prognosis than the RS1 group, suggesting the hypoxic status of HCC may be associated with resistant mechanism to TACE treatment, which is consistent with previous findings (39–41). Improving the hypoxic status in tumors is potentially one of the methods to improve the therapy effect and this mechanism should be analyzed in more detail.

Our study has some limitations. First, the number of patients with middle-stage HCC was relatively small. Second, the study was conducted retrospectively. However, we used CT images from four centers, and all the CT images were normalized before extracting pyradiomics features to develop a robust predictive model. Multi-center model analysis also showed robust predictive performance. However, the model still needs to be validated in larger prospective studies. Third, we trained and validated all the pyradiomics signatures in four medical centers. Extracting features from ROI images may have issues in reproducibility. In this study, we evaluated the reproducibility of pyradiomics and found a good agreement (ICC>0.75), markedly improving the predictive model’s robustness. Future studies should develop an automatic segmentation model for liver tumors and minimize the discrepancies among pyradiomics features.

In conclusion, the machine learning model based on pyradiomics features from 3D-CT images is a noninvasive yet highly accurate model for predicting the initial response and prognosis to TACE in patients with HCC. Thus, it may be a feasible tool for identifying patients who will benefit from TACE. The association between the pyradiomics model and cancer-related signaling pathways might help clinicians further understand the internal mechanism of machine learning. Finally, this radiology method could be used to improve the accuracy in clinical decision-making for other types of malignant tumors.

## Data availability statement

For study involving human sample: The original contributions presented in the study are included in the article/**Supplementary Material**. Further inquiries can be directed to the corresponding author. For study involving TGCA data: The datasets presented in this study can be found in online repositories. The names of the repository/repositories and accession number(s) can be found in the article/**Supplementary Material**.

## Ethics statement

The studies involving human participants were reviewed and approved by Nanfang Hospital, the Second Affiliated Hospital of Gui Zhou Medical University, and Sun Yat-sen University Cancer Center. The patients/participants provided their written informed consent to participate in this study.

## Author contributions

(I)Conception and design: JP. (II) Administrative support: None. (III) Provision of study materials or patients: JH and JP. (IV) Collection and assembly of data: JP, JZ, and FL. (V) Data analysis and interpretation: JP and JH. (VI) Manuscript writing: All authors and (VII) Final approval of manuscript: All authors.

## Funding

This work was supported by the Science and Technology Fund Project of Guizhou Provincial Health Commission [gzwjkj2019-1-077], Qian Dong Nan Science and Technology Program [qdnkhJz2019-026], Open Funds of State Key Laboratory of Oncology in South China [HN2020-02], Qian Dong Nan Science and Technology Program [qdnkhJz2020-013], the Guizhou Medical University 2018 academic new talent cultivation and innovation exploration project [Grant No. 20185579-X], Science and Technology Foundation of Guizhou Province [Grant No. Qian ke he ji chu-ZK 2021, yi ban 454], and National Nature Science Foundation of China (Grant Nos. 82060327).

## Conflict of interest

The authors declare that the research was conducted in the absence of any commercial or financial relationships that could be construed as a potential conflict of interest.

## Publisher’s note

All claims expressed in this article are solely those of the authors and do not necessarily represent those of their affiliated organizations, or those of the publisher, the editors and the reviewers. Any product that may be evaluated in this article, or claim that may be made by its manufacturer, is not guaranteed or endorsed by the publisher.

## Abbreviations

AFP, alpha-fetoprotein; AIC, Akaike information criterion; AUC, area under the curve; BCLC, Barcelona Clinic Liver Cancer; CI, confidence interval; CR, complete response; CT, computed tomography; DFS, disease-free survival; HCC, Hepatocellular carcinoma; HR, hazard ratio; ICCs, inter-correlation coefficients; KEGG, Kyoto Encyclopedia of Genes and Genomes; LASSO, least absolute shrinkage and selection operator; OS, overall survival; PD, progressive disease; PFS, progression-free survival; PR, partial response; PRS, pyradiomics score; ROC, receiver operating characteristic; ROI, regions of interest; SD, stable disease; TACE, trans-arterial chemoembolization; TCGA, The Cancer Genome Atlas; NCCN, National Comprehensive Cancer Network; MRI, magnetic resonance imaging.
