# Supplementary material for: Development and validation of a pyradiomics signature to predict initial treatment response and prognosis during transarterial chemoembolization in hepatocellular carcinoma
Source: Front Oncol. 2022 Oct 17;12:853254. doi: 10.3389/fonc.2022.853254 (PMC9618693; doi:10.3389/fonc.2022.853254)
Supplement: Supplementary file 1 [file DataSheet_1.docx]

Supplementary Material

**CT image acquisition**

Contrast-enhanced CT (CECT) images were acquired at three hospitals, using the following multi-detector row CT (MDCT). The Second Affiliated Hospital used SOMATOM (Siemens Medical Systems, Erlangen, Germany) scanner, Nanfang and Sun Yat-Sen University Cancer Center used Brilliance iCT 256 (Philips Healthcare, Cleveland, OH) scanner. Scanning parameters used in this study as following respectively: tube voltage, 120 kVp; detector collimation, 64×0.6 and 128×0.625 mm; field of view, 250-400 mm; pixel size, 512×512; rotation time, 0.5 s; slice interval, 0 mm; slice thickness, 5 mm; reconstructed section thicknesses, 1 mm. CECT images were acquired after injection of 1.0 mL/kg contrast material (Ultravist 370, Bayer Schering Pharma, Berlin, Germany) into the antecubital vein at a rate of 2.0–3.0 mL/s using a power injector (Ulrich CT Plus 150, Ulrich Medical, Ulm, Germany); this was followed by a saline flush (20mL). Triphasic (hepatic arterial, portal venous, and delayed phase) CT images were acquired at 30, 60, and120 s after contrast material injection, respectively. The slice thickness of reconstructed arterial and portal venous phase images was 5 mm.

## Supplementary Tables

| **Table S1. Predictive performance of the pyradiomics signature for treatment response** | | | | |
| --- | --- | --- | --- | --- |
| **Groups** | **Accuracy**  **(95% CI)** | **Sensitivity**  **(95% CI)** | **Specificity**  **(95% CI)** | ***P* value** |
| Discovery set | 0.91 (0.86-0.96) | 0.89 (0.80-0.95) | 0.82 (0.69-0.91) | <0.001 |
| Validation set 1 | 0.90 (0.84-0.95) | 0.78 (0.67-0.87) | 0.90 (0.78-0.96) | <0.001 |
| Validation set 2 | 0.91 (0.83-0.98) | 0.93 (0.79-0.99) | 0.78 (0.54-0.93) | <0.001 |

Abbreviations: CI, confidence interval

| **Table S2. Predictive performance of the pyradiomics model by subgroup** | | | | |
| --- | --- | --- | --- | --- |
| **Subgroups** | **Accuracy**  **(95% CI)** | **Sensitivity**  **(95% CI)** | **Specificity**  **(95% CI)** | ***P* value** |
| Sex |  |  |  |  |
| Female | 0.96 (0.92-1.00) | 0.95 (0.75-0.99) | 0.80 (0.58-0.94) | <0.001 |
| Male | 0.89 (0.86-0.93) | 0.80 (0.71-0.87) | 0.86 (0.80-0.91) | <0.001 |
| Age (years) |  |  |  |  |
| ≤60 | 0.89 (0.85-0.93) | 0.81 (0.73-0.87) | 0.84 (0.74-0.91) | <0.001 |
| >60 | 0.92 (0.87-0.97) | 0.88 (0.77-0.95) | 0.83 (0.69-0.92) | <0.001 |
| Child–Pugh classification |  |  |  |  |
| A | 0.90 (0.87-0.94) | 0.90 (0.84-0.94) | 0.75 (0.66-0.83) | <0.001 |
| B | 0.90 (0.80-1.00) | 0.93 (0.79-0.99) | 0.83 (0.58-0.96) | <0.001 |
| AFP (ng/mL) |  |  |  |  |
| ≤20 | 0.90 (0.86-0.93) | 0.88 (0.82-0.92) | 0.78 (0.68-0.85) | <0.001 |
| >20 | 0.93 (0.86-1.00) | 0.96 (0.83-0.99) | 0.80 (0.58-0.94) | <0.001 |
| Tumor size (cm) |  |  |  |  |
| ≤10 | 0.87 (0.82-0.92) | 0.84 (0.74-0.91) | 0.78 (0.69-0.85) | <0.001 |
| >10 | 0.90 (0.83-0.97) | 0.90 (0.83-0.95) | 0.79 (0.57-0.92) | <0.001 |
| Tumor number |  |  |  |  |
| ≤3 | 0.90 (0.85-0.95) | 0.90 (0.83-0.95) | 0.79 (0.67-0.88) | <0.001 |
| >3 | 0.91 (0.87-0.96) | 0.90 (0.80-0.96) | 0.80 (0.70-0.87) | <0.001 |
| Note: *P* value is derived from the difference between the discovery data set and the two validation data sets.  Abbreviations: AFP, alpha-fetoprotein; CI, confidence interval; CR, complete response; PR, partial response; SD, stable disease; PD, progressive disease | | | | |

## Supplementary Figures


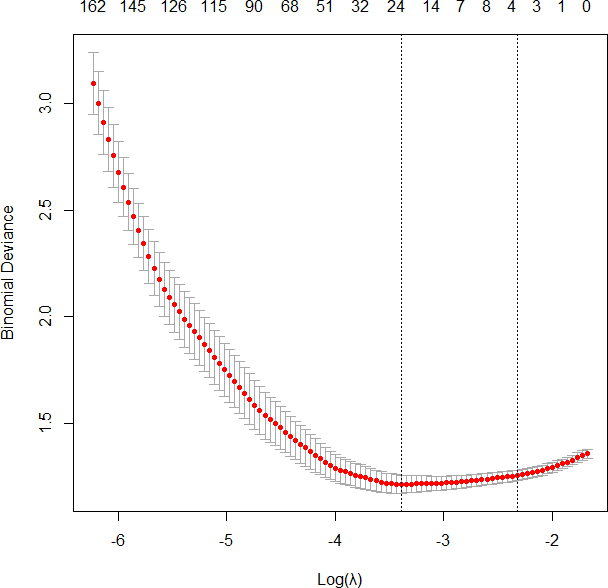


**Supplementary Figure 1.** LASSO coefficient analysis of 1,110 pyradiomic features. A total of 24 coefficients are chosen using 5-fold cross-validation, shown as the vertical line presented in the plot.

Abbreviations: LASSO, least absolute shrinkage and selection operator algorithm


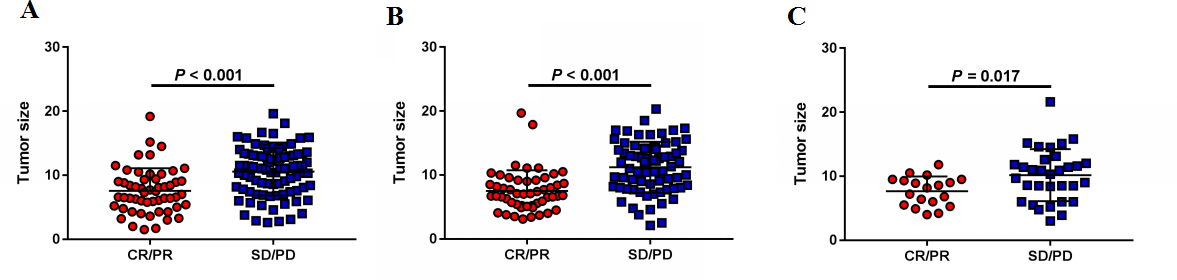


**Supplementary Figure 2.** Comparison of tumor size between the CR/PR and SD/PD groups in the discovery set (**A**), validation set 1 (**B**) and validation set 2 (**C**).

Abbreviations: CR, complete response; PR, partial response; SD, stable disease; PD, progressive disease


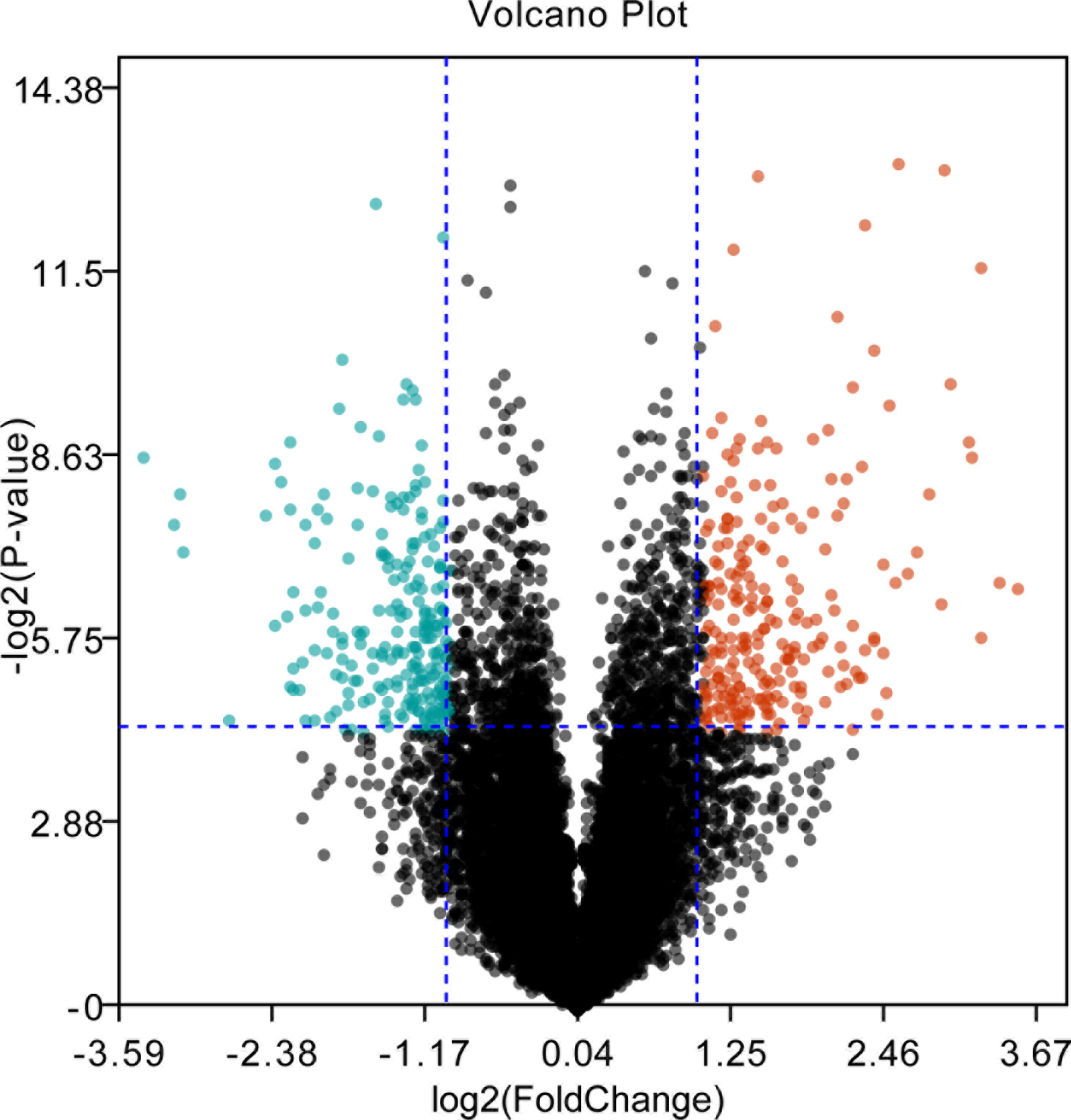


**Supplementary Figure 3.** Volcano plots for mRNA gene expression profiling in the RS1 and the RS2 groups.


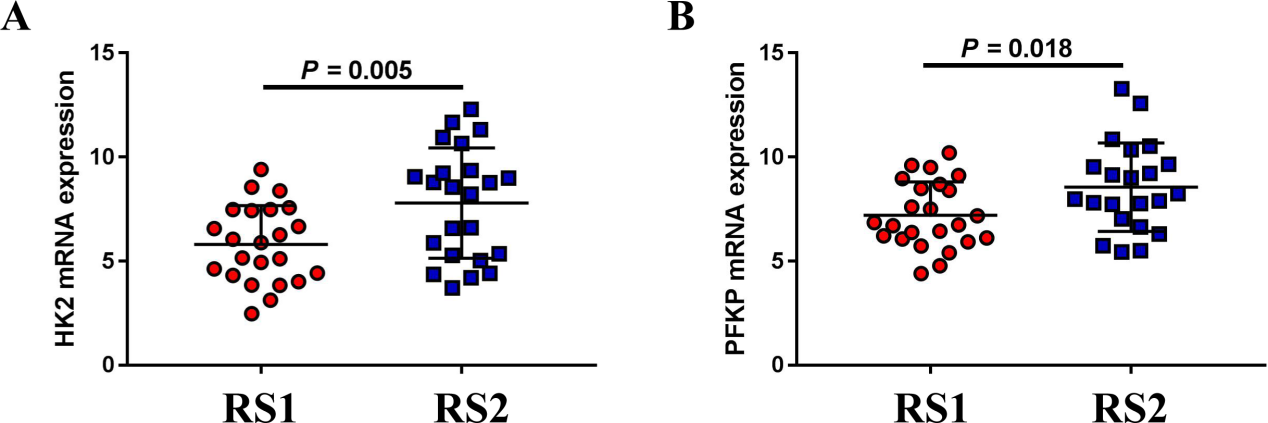


**Supplementary Figure 4.** Relative mRNA expression levels of *HK2* (A) and *PFKP* (B) between the RS1 and RS2 groups.
